# Emergent Repair of a Perforated Giant Duodenal Ulcer in a Patient With an Unmanaged Ulcer History

**DOI:** 10.7759/cureus.12198

**Published:** 2020-12-21

**Authors:** Eli B Eisman, Nicole C Jamieson, Rashona A Moss, Melina M Henderson, Richard C Spinale

**Affiliations:** 1 College of Osteopathic Medicine, Michigan State University, East Lansing, USA; 2 General Surgery, Garden City Hospital, Garden City, USA; 3 Internal Medicine, Garden City Hospital, Garden City, USA; 4 Obstetrics and Gynecology, Garden City Hospital, Garden City, USA

**Keywords:** duodenal ulcers, complicated peptic ulcer disease, gastrointestinal perforation, duodenal ulceration, perforated duodenal ulcer, chronic ulcer, graham patch repair

## Abstract

Giant duodenal ulcers (GDUs) are full-thickness disruptions of the gastrointestinal epithelium greater than 3cm in diameter. The significant size and disease chronicity lead to deleterious outcomes and high mortality risk if ulcer progression is not halted. While still prevalent in developing countries, GDUs are increasingly rare in industrialized nations. Here, we present the case of an 82-year-old woman with perforated GDU requiring emergent surgical intervention complicated by prior duodenal surgery requiring a previously unreported triple-layered omental patch. Discussion of this technique and novel approaches to GDU repair ensue.

## Introduction

Giant duodenal ulcers (GDUs) were first characterized in 1931, though initially difficult to diagnose on barium study due to the mistaken identification of ulcers as deformed duodenal caps [1]. Nussbaum and Schusterman codified GDUs as full-thickness ulcers greater than 2cm, usually involving but not limited to the duodenal bulb [2]. Gupta et al. revised that scheme classifying ulcers 1-3cm as large and greater than 3cm as a giant [3]. The advent of H2-receptor antagonists (H2I), as well as increasingly routine endoscopic evaluation, led to a marked decline of GDU diagnosis beginning in the 1990s [4]. GDUs are closely associated with typical risk factors for peptic ulcer disease (PUD) with a slightly stronger correlation with aspirin and nonsteroidal anti-inflammatory drug (NSAID) use rather than Helicobacter pylori (H. pylori) infection [5]. The lifetime prevalence of duodenal ulcers is 11-20% for men and 8-11% for women and of those diagnosed, 5% will eventually necessitate operative intervention [6,7]. Worsening outcomes and risk of duodenal ulcer perforation are inversely correlated with the length of time before management is implemented [8]. The high risk of perforation, 25% if the ulcer is either large or giant, and 10% global mortality risk, should prompt initiation of medical management aimed at halting disease progression when symptoms first become apparent [3,4,9,10].

PUD with duodenal involvement presents with a constellation of symptoms most commonly epigastric and right upper quadrant pain that may include radiation to back. Melena, hematochezia, and hematemesis can also be observed. Signs of chronic malnutrition, including anemia, weight loss, and cachexia, are typical. The current standard of care is medical management with proton pump inhibitors (PPI) replacing H2Is as first-line treatment and triple or quadruple therapy for treating H. pylori infections [11,12]. Surgical repair is indicated if conservative management fails or if red-flag symptoms such as perforation or bleeding are demonstrated [13]. GDU repair carries a high perioperative risk, with improved outcomes if the ulcer is detected early [14]. Patients undergoing surgical repair with a poor medical history and compounding comorbidities, tend to be hemodynamically unstable at the time of intervention with a perioperative mortality rate of 10-65%, supporting the role for aggressive medical or elective surgical management before perforation occurs [15,16].

## Case presentation

JF is an 82-year-old Caucasian female with a past medical history of two cerebrovascular accidents in the past year, coronary artery disease status-post stent placement, recurrent gastric ulcers, hypertension, hyperlipidemia, hypothyroidism, and Alzheimer’s type dementia. She initially presented to the emergency department with a chief complaint of asthenia and an acute onset burning sensation diffusely in the right upper quadrant. She also stated that she had left shoulder and hip pain following an unwitnessed fall in the shower 24 hours prior, hitting head though denying the loss of consciousness. Further history obtained from her son indicated that at baseline JF ambulates with a walker and is competent to manage her acts of daily living. However, the patient had been complaining of weakness for the preceding 2-3 days and had difficulty rising from bed that morning in addition to abrupt onset back pain, difficulty ambulating, and slurred speech.

**Figure 1:**
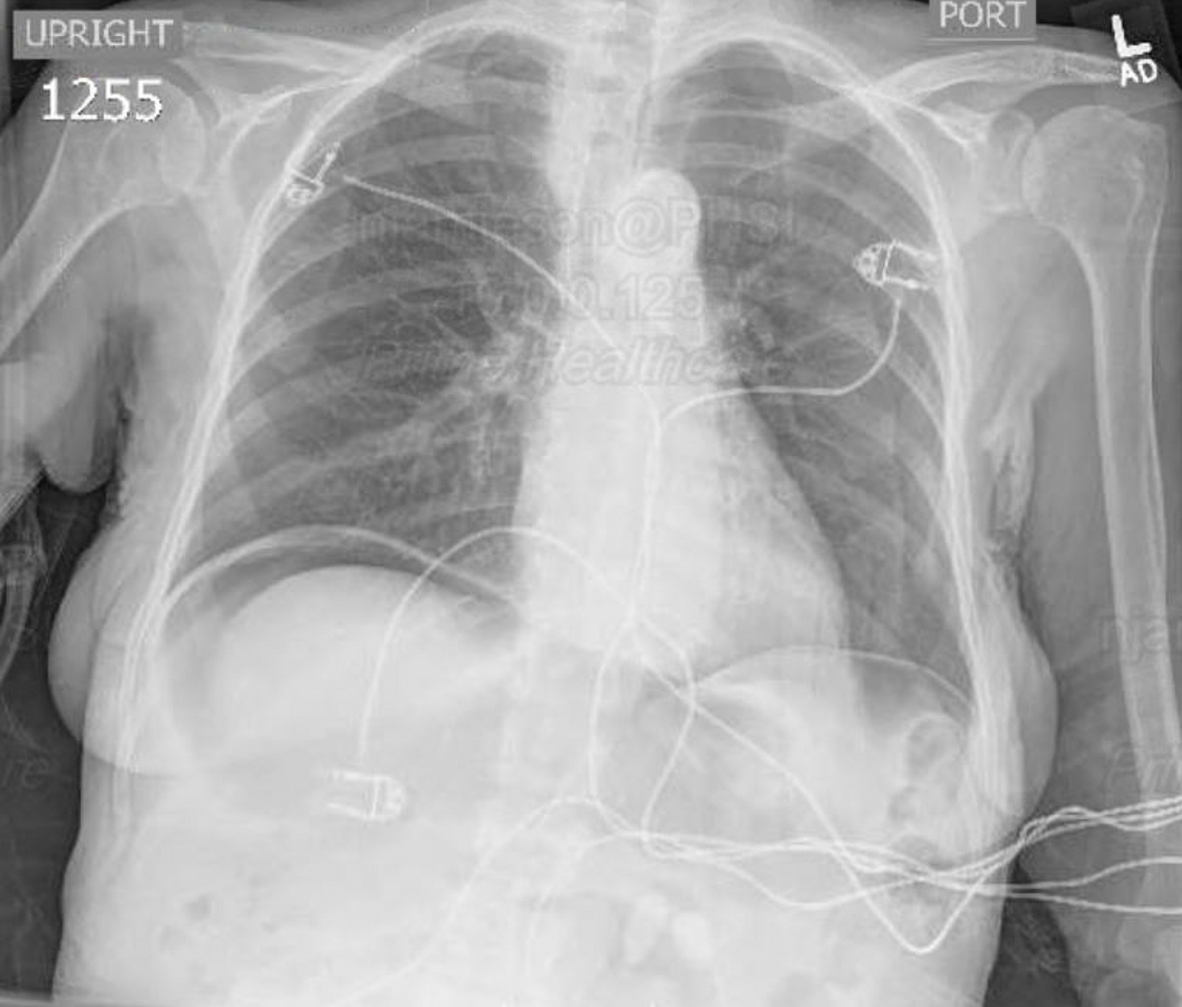
Chest X-ray demonstrating pneumoperitoneum.

Evaluation in the ED demonstrated a hypothermic, 95.7F rectal, bradycardic, 51 bpm, hypotensive, BP 88/35, tachypneic, 36 respirations per minute, female in acute distress with diffuse skin mottling present symmetrically over the lower extremities. NIH stroke scale was determined to be 8, at which time the stroke protocol was activated. CT brain was significant for a right occipital hypodense lesion that could not be ruled out for acute processes. Preliminary laboratory results were notable for hyperkalemia, leukocytosis, and elevated troponins, with EKG demonstrating ST elevations in leads II, III. Chest X-ray demonstrated pneumoperitoneum (Fig. 1) and the patient was admitted to the surgery service for emergent exploratory laparotomy. While in the ED, the patient was given a 1-litre bolus of Lactated Ringers and cardioverted with atropine for symptomatic bradycardia which yielded an appropriate response. Given the patient’s acutely critical condition and impending respiratory failure, the patient was intubated in the ED for airway protection before being transported to the operating room. Additionally, the patient’s blood pressure was stabilized with Levophed and empiric Rocephin, Flagyl, and Unasyn were administered.

The patient was given a pre-operative diagnosis of septic shock and perforated viscus. The abdominal cavity was entered through a midline incision and electrocautery, revealing a voluminous amount of bile as well as undigested food particulate present in all four quadrants. The fluid was sampled for cultures and removed via suction. Once unobscured, dense adhesions between the stomach, liver, and omentum as well as anterior surface of the duodenum were identified. Additionally, the proximal transverse colon was significantly bile stained and without evidence of acute perforation. Adhesiolysis and takedown of the Falciform ligament for improved visualization exposed a large anterior duodenal ulcer immediately distal to the pylorus in the first portion of the duodenum. Prior sutures on the posterior aspect of the duodenal lumen were also noted from a previous surgery that was performed for GI haemorrhage. An extensive four-quadrant washout using warm saline was performed until irrigation was noted to be clear.

**Figure 2:**
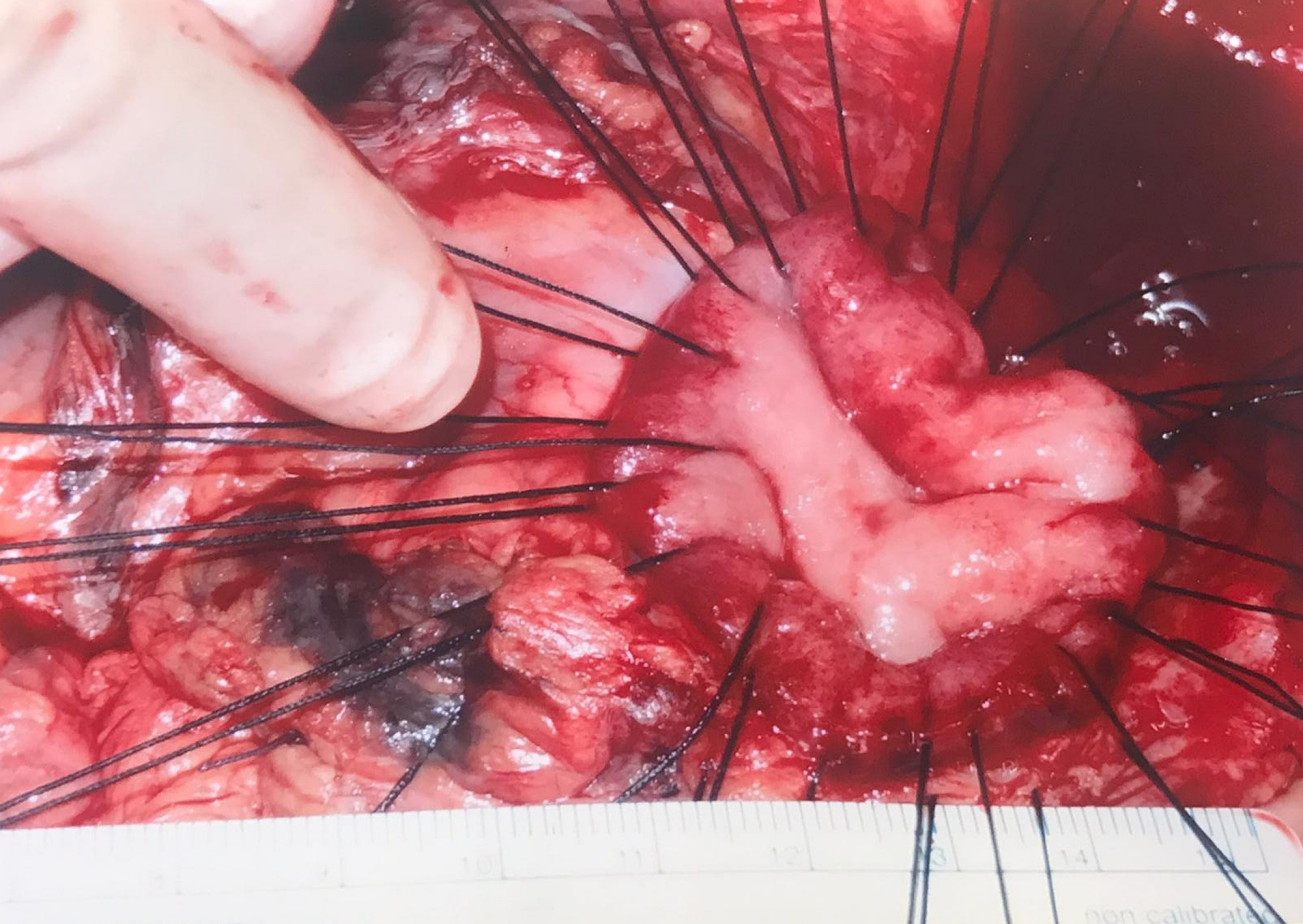
Giant duodenal ulcer with full thickness bites along ulcer perimeter.

**Figure 3:**
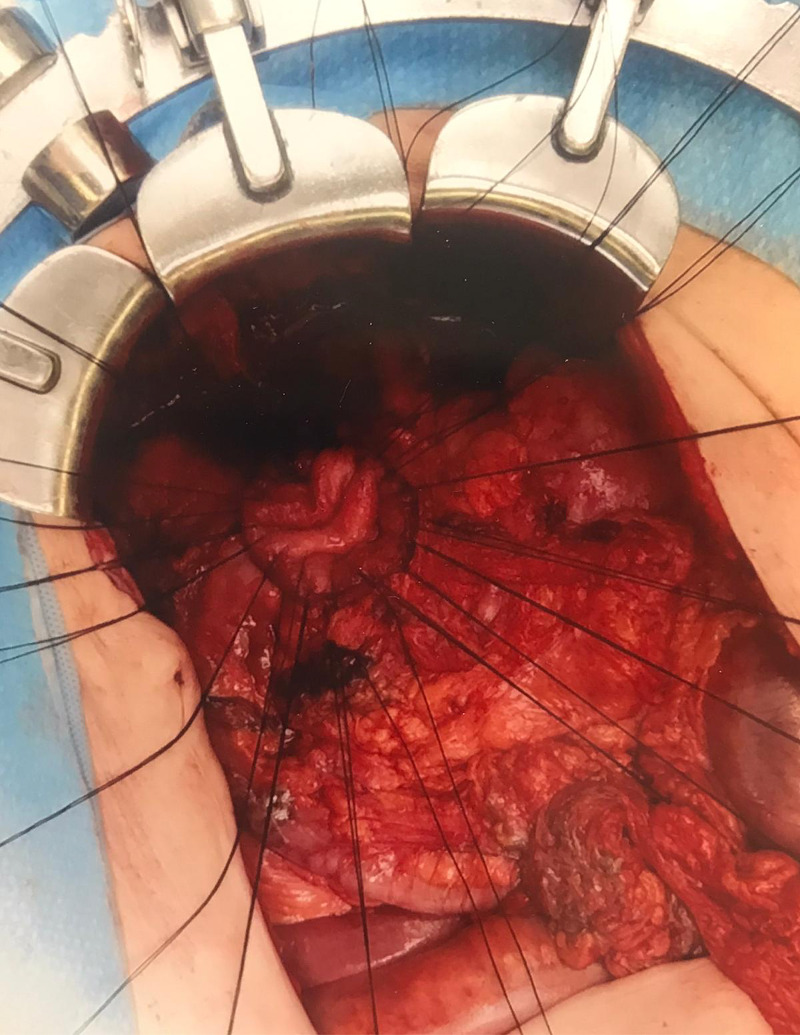
Duodenal ulcer with silk sutures placed.

**Figure 4:**
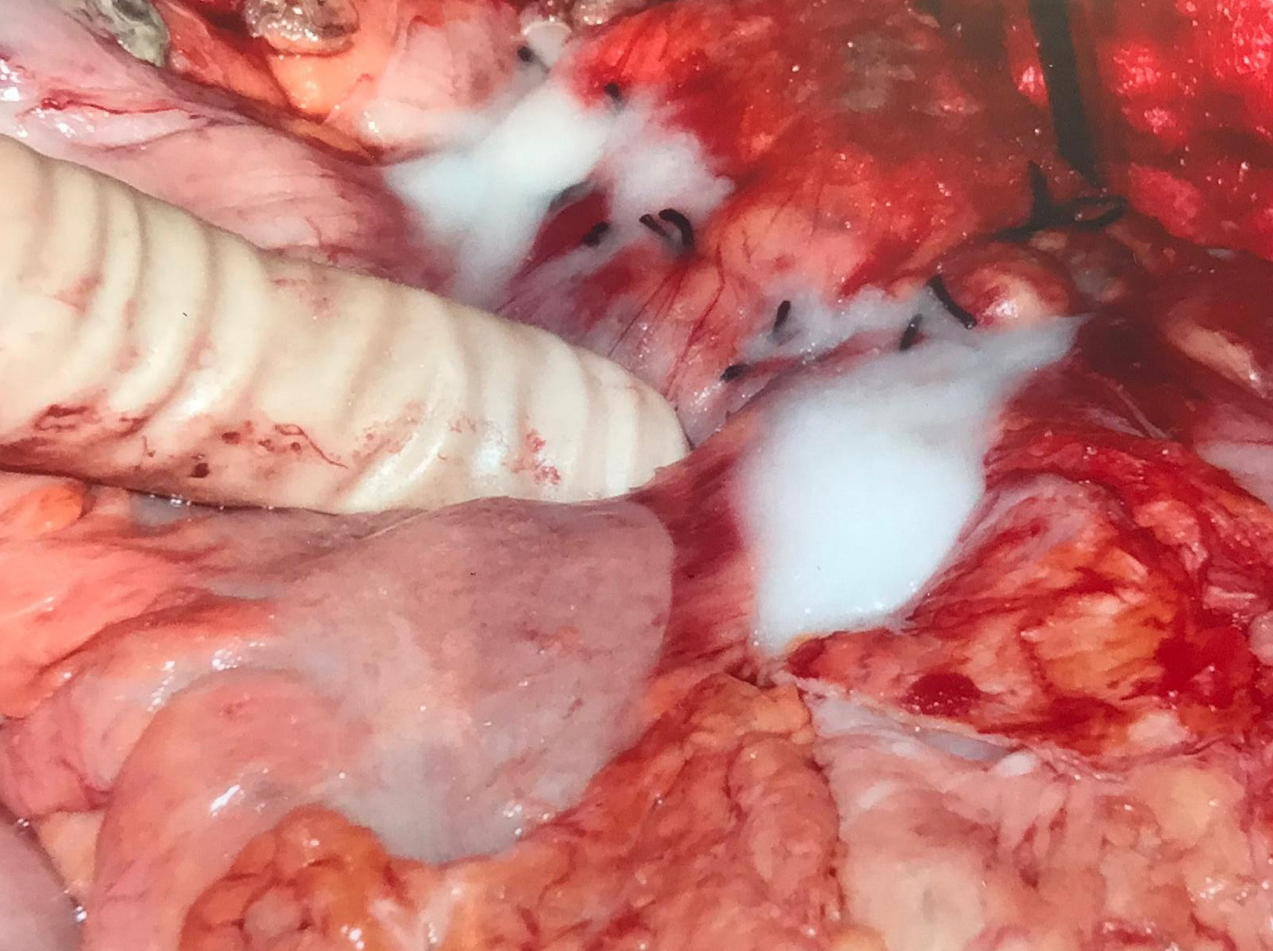
Modified triple layer omentum Graham patch with fibrin glue.

Evaluation of the ulcer revealed a 4.0 cm defect with 1.5 cm raised, thickened, hyperemic edge (Fig. 2, 3). With the size, extensively raised borders, apparent prior duodenal surgery, and patient acuity in mind, a tripartite Graham patch arranged circumferentially, ensuring maximal thickness, was opted for the closure of the perforation. This was performed using 0-silk suture in a simple interrupted fashion. Full-thickness bites from serosa to mucosa were taken around the entire perimeter of the exposed duodenal ulcer with initial placement in the duodenum. As the omentum was noted to be diminutive, it was thrice-folded upon itself and bites through the triple-layered omentum were taken and parachuted into place as a Graham patch. Once the anchoring sutures were completed and tied, fibrin glue was applied to both the anterior and posterior surfaces of the repair (Fig. 4). Reinspection of the liver necessitated further hemostatic control, attained with Floseal, Surgicel, and electrocautery for inadvertent injury during adhesiolysis. Jackson-Pratt (JP) drains were placed in the left upper quadrant in the splenorenal gutter, left lower quadrant in the pelvic gutter, right upper quadrant in the foramen of Winslow, and right lower quadrant in the hepatorenal gutter and sutured in place with 3-0 nylon. The linea alba and peritoneum were closed in a simple running fashion and the skin was approximated using a skin stapler with overlying sterile dressing. The patient was sent to the ICU in critical condition and remained on vasopressors for hemodynamic support and total parental nutrition started post-operatively. Additionally, a nasogastric tube placed by the anesthesiologist during the operation was set to low intermittent suction and an intravenous PPI was initiated.

JF’s post-operative course in the ICU was complicated by non-ST-elevation myocardial infarction (NSTEMI) Type II, septic shock with progression to DIC requiring four units of packed red blood cells, six units of plasma, and three units of platelets. Acute renal failure followed shortly with little improvement in urine output with the administration of Bumex. Eventual Quinton catheter placement and multiple rounds of dialysis were required, with Levophed necessary to maintain MAP above 70mmHg during treatment. On post-operative day seven enteral nutrition was started at a rate of 10cc/hour. On post-operative day eight the patient was noted to have tolerated the tube feedings, and they were therefore advanced to a goal rate. The patient also received multiple bronchoscopy procedures for mucus plugs and failed multiple rounds of ventilator weaning trials. During this time the JP drains remained in place and continued to drain serosanguinous fluid, while the incision remained clean, non-erythematous, dry, and intact. On post-operative day 13, the patient was noted to have bilious drainage from the right upper quadrant JP drain and presumed leak from the repaired ulcer. Enteral feedings were held and nasogastric tube replaced and set to low intermittent suction. Discussion of the necessity for eventual tracheostomy and percutaneous endoscopic gastrostomy tube had previously been initiated with the family. In light of the recent leak, need for continual hemodialysis, and the patient’s multiple other comorbidities, the family elected to make the patient comfort measures only and a terminal wean was performed, the patient expired shortly after.

## Discussion

The frequency of giant duodenal ulcers has significantly waned since the 1990s with the introduction of H2-receptor antagonists. This rate declined further with the advent of proton pump inhibitors, decreasing the incidence of H. pylori infection, and increasing access to diagnostic esophagogastroduodenoscopy. However, GDUs remain a non-rare occurrence in developing countries and populations without routine care in industrialized nations [4,17]. The complexity of the surgical repair and tendency for post-operative complications is determined by the size of the ulcer, edematous or necrotic margins, high intraluminal pressure, and underlying comorbidities. Options for repair include omentopexy, omental plugging, partial gastrectomy, jejunal serosa patch, jejunal pedalled graft, proximal gastrojejunostomy, and pyloric exclusion [3,18,19].

In their retrospective analysis of 162 perforated duodenal ulcers, Gupta and colleagues report only two GDUs, or 1.23% of all cases over 24 months, indicating the exceptional rarity of the pathology [3]. Their investigation demonstrated a statistically significant lengthening of hospital stay, increased leak rate, and morbidity when contrasting perforated small and large duodenal ulcers. At the same time, mortality rates increased from 5.74% to 15.79% to 50%, for small, large, and giant ulcers, respectively. The paucity of patients with GDU prohibited an extensive comparison with the smaller classes. However, we hypothesize increased perioperative morbidity would be expected in patients when comparing small and giant ulcers. 

In a recent report evaluating a novel technique aimed at minimizing post-surgical leak and abating the high mortality incidence associated with the perforated GDU repair, Lal et al. address non-emergent surgical management in patients with this diagnosis [15]. In their work, 40 demographic and pathology matched patients were considered, 20 control receiving standard of care treatment of either pedicled Cellan-Jones patch or Graham patch, and 20 receiving an experimentally controlled tube duodenostomy. Post-surgical complications were experienced in both the control and experimental groups and included wound infection, dehiscence, pneumonitis, intra-abdominal sepsis, and death. Notably, post-operative characteristics were significantly reduced for those patients receiving the experimental treatment. For 19/20 patients undergoing duodenostomy, they were discharged from the hospital within 12-20 days following the surgery, while all of the patients receiving the control treatment remained beyond 25 days. Remarkably, the 30-day mortality for the control group was 13/20. At the same time, the single death reported in patients receiving the experimental treatment was attributable to fulminant pulmonary tuberculosis and not sequelae of the surgery. The authors conclude, however, that this technique is technically challenging and may not be suitable for an emergent setting. In response to this paper, Malangoni maintained GDU repair requiring emergency surgery should be completed by Graham patch, either open or laparoscopically, with or without definitive ulcer operation of vagotomy with pyloroplasty or partial gastrectomy [20]. He continued, suggesting that Lal et al. do not risk stratify by organ systems nor consider local inflammatory sequelae seen in a perforated ulcer, contrasted with duodenal injury, as proposed. Additionally, coexisting comorbidities that could lead to pre-operative complications require evaluation beyond the simple closure and decompression as detailed.

## Conclusions

Notably, in the case of JF, her prior ulcer surgery prevented resection, and the enormity of the ulcer prevented serosal patching, as the intestinal diameter was barely half the ulcer crater diameter. Moreover, when folded, a double-layered omentum was insufficient for patching requiring a previously unreported triple-layered approach. Despite reinforcement, leakage from the surgical site was observed, and though a common complication of this procedure, the risk was likely increased resulting from the patient’s underlying comorbidities and baseline instability. If the patient had been hemodynamically stable during the surgery, a feeding Jejunostomy tube could have been placed to provide the patient with enteral feeds distal to the repair earlier during the post-operative period. Finally, the giant diameter of the ulcer crater along with fibrosis from prior surgery rendered it technically impossible to do tube duodenostomy closure even with the largest tube available. 
